# Mathematical Characterization of Protein Transmembrane Regions

**DOI:** 10.1155/2013/607830

**Published:** 2013-04-15

**Authors:** Amrita Roy Choudhury, Nikolay Zhukov, Marjana Novič

**Affiliations:** ^1^Laboratory of Chemometrics, National Institute of Chemistry, Hajdrihova 19, 1001 Ljubljana, Slovenia; ^2^Faculty of Mathematics, Informatics, and Mechanics, University of Warsaw, Banacha 2, 02-097 Warszawa, Poland

## Abstract

Graphical bioinformatics has paved a unique way of mathematical characterization of proteins and proteomic maps. The graphics representations and the corresponding mathematical descriptors have proved to be useful and have provided unique solutions to problems related to identification, comparisons, and analyses of protein sequences and proteomics maps. Based on sequence information alone, these descriptors are independent from physiochemical properties of amino acids and evolutionary information. In this work, we have presented invariants from amino acid adjacency matrix and decagonal isometries matrix as potential descriptors of protein sequences. Encoding protein sequences into amino acid adjacency matrix is already well established. We have shown its application in classification of transmembrane and nontransmembrane regions of membrane protein sequences. We have introduced the dodecagonal isometries matrix, which is a novel method of encoding protein sequences based on decagonal isometries group.

## 1. Introduction

With the advent of modern and faster experimental technologies, a large amount of sequences and proteomics data are produced every day. Combined with the genomic data, this forms a vast and ever-growing repository of information. However, to utilize this data in order to get an insightful knowledge of the biological systems and develop better pharmaceutical facilities, one needs to perform a detailed analysis of the data generated. The new age computational methods provide fast, accurate, precise analysis of the genomics and proteomics data. Therefore, there is a need to characterize biological sequences and data in mathematical formats that can be easily manipulated using the computational methods. 

Mathematical graphs and matrices have been successfully utilized in representing, characterizing, and analyzing biological sequences. Even though the graphical representation of DNA was initiated around 25 years ago, graphical methods to represent protein sequences and proteomics maps emerged only recently [1, 2]. The delay is owed to the increase in complexity and associated arbitrariness in assigning and representing the 20 natural amino acids, which can be done in 20 factorial ways. The initial methods proposed to graphically represent the protein sequences are Magic Circle [3] and Starlike graphs [4]. Both representations are associated with no loss of information and offer novel local alignment methods using Euclidean distances between corresponding amino acids in the graphical representation. To characterize protein sequences numerically, structural matrices like D/D matrix and Line Distance matrix are developed from 2D graphs of proteins [3]. Invariants of such matrices, for example, eigenvalues, matrix diagonals, row sums, and so forth, further serve as numerical representation of the proteins. 

Amino acid adjacency matrix (AA matrix) [5] is a matrix representation of protein sequences leading to mathematical characterizations. The protein sequence, in this case, is directly translated into the matrix form without the intermediate graphical representation. To represent a protein sequence mathematically, any invariant of its amino acid adjacency matrix can be used. Here, we have considered the row sum invariant of the amino acid adjacency matrix to numerically characterize protein sequences. 

We have also introduced the encoding of transmembrane regions from the perspective of decagonal isometries group (D_10_) into decagonal isometries matrix (DIM). The DIM is then transformed into 20-dimensional vector, which is then used to represent and numerically characterize the protein sequences.

It is important that the code of amino acid sequence is of uniform dimension, regardless of the length of the protein segment. We have adopted this criterion for both representations applied in transmembrane segments classification study. The amino acid sequence was the only information source for AA matrix and DIM representations developed or applied in this work. Conversely, other features associated with amino acids and proteins are often used in protein structure-property studies, such as secondary structure propensity, hydrophobicity, polarizability, solvent accessibility, normalized van der Waals volume, and polarity enrichment scores in case of the analysis and prediction of the metabolic stability of proteins [6]. The criterion of uniform dimensionality has been followed also in the study of single amino acid polymorphisms (SAPs), which is accounted for the majority of human inherited diseases. Each SAP is represented by 472 features including sequential, structural, and network features, the latter being the most influential [7]. However, in our study we have shown that the protein sequence alone if encoded into a suitable uniform representation vector enables us to build a successful classification model that separates transmembrane regions of a protein from nontransmembrane ones. 

Transmembrane proteins pass through the complete biological membrane and perform vital functions to maintain the normal cell physiology. They are also very important as drug targets. These proteins are therefore of immense interest from both the academic and pharmaceutical point of views [8]. Despite the importance and interest, the vast majority of the transmembrane protein space remains unexplored due to experimental difficulties. Not all the transmembrane proteins, hypothesized to be present, are yet reported and sequenced. Only few of the known transmembrane proteins have their structures resolved to atomic details. In this work, we have focused on representing the transmembrane protein sequences numerically in order to develop novel transmembrane protein sequence analysis methods. Both the amino acid adjacency matrix and decagonal isometries matrix, explained in this work, are applied towards characterizing protein transmembrane regions and distinguishing them from the nontransmembrane regions.

## 2. Materials and Methods

### 2.1. Amino Acid Adjacency Matrix

**Figure 1:**
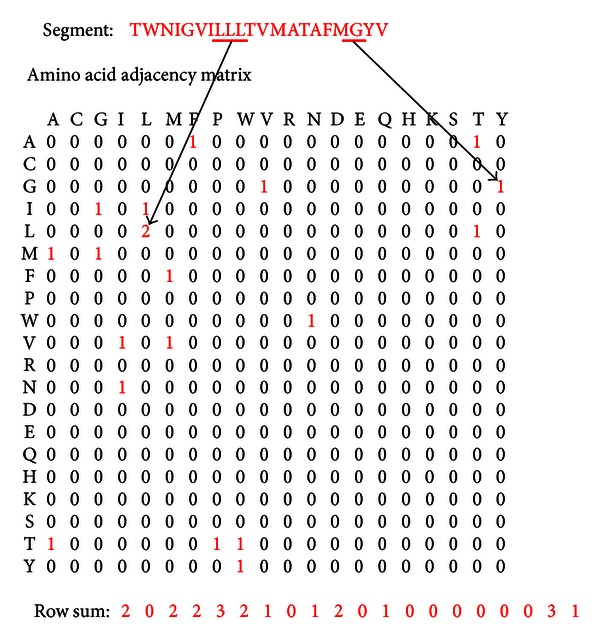
Amino acid adjacency matrix. The 20 × 20 matrix presenting the amino acid adjacency and abundance information in the given sequence. The nonzero elements show the number of times that corresponding amino acids are present adjacent to each other. The 20-dimensional row sum vector is used as a descriptor to numerically characterize the protein sequence.

The amino acid adjacency matrix (AA matrix) is a nonsymmetric matrix that presents the adjacency information of the 20 natural amino acids in the given protein sequence [5]. It is a 20 × 20 matrix with the rows and columns labeled with the 20 amino acids (Figure 1). Each position in the matrix represents the number of times the corresponding amino acids are adjacent in the given sequence; that is, value of matrix element (*i*, *j*) depends on the number of times amino acid in row *i* is followed by amino acid in column *j* in the given sequence. For example, in Figure 1, amino acids G and Y are adjacent to each other only once, and hence the value of element (G, Y) is 1. Similarly the value of (L, L) is 2 as L occurs as its own first neighbor twice. The 400 matrix elements thus record the adjacencies of amino acids and their abundance in a given protein sequence. 

As the matrix invariants do not depend on the labeling of the matrix, an arbitrary ordering of the amino acids is sufficient when one is interested in bringing out the characteristic features of a given sequence and in differentiating between sequences. In our work, we arbitrarily choose the amino acid to be in the following order: A, C, G, I, L, M, F, P, W, V, R, N, D, E, Q, H, K, S, T, Y.

It must be noted that the amino acid adjacency matrix is essentially different from both the GRANTHAM matrix [9] and the neighbor-dependent amino acid propensity [10]. The GRANTHAM matrix predicts the effect of amino acid substitution based on chemical properties. In our case, the matrix is independent of amino acid properties and does not reflect substitution effects. The matrix records the adjacency and not the propensity of the amino acid to be present at a particular structural location. In this study, we have considered only the first neighbor of a particular amino acid position. We have implemented the AA matrix representation of the transmembrane segments of membrane proteins in building transmembrane region prediction model. It must be noted that the transmembrane segments are around 20 residues in length. The short segments therefore result in sparse AA matrices. The matrix elements with zero values denote the absence of the corresponding amino acid pairs in the sequence. If a particular amino acid is not present in the given sequence, the corresponding row and column have all entries zero. In the given example (Figure 1), the amino acids C, P, R, D, E, Q, H, K, S are not present. One must also note that the last residue of the sequence is not shown in the adjacency matrix, as it has no adjacent residue to its right.

The important aspect of using matrix presentation of amino acid adjacencies is that it enables a concise numerical characterization of a protein segment by matrix invariants. The simplest characterization of protein can be presented as a 20-dimensional row sum vector that lists the abundance of the 20 amino acids except for the last residue of the protein segment, as explained in the previous paragraph.

### 2.2. Decagonal Isometries Matrix

**Figure 2:**
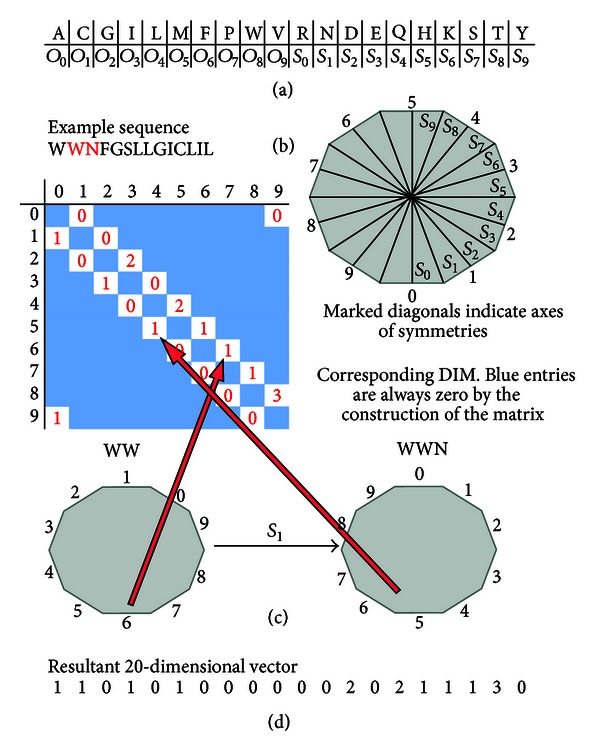
Decagonal isometries matrix D_10_. (a) and (b) show correspondance between group elements and amino acids and indicate the initial step of coding. Decagons below show the explicit transformation for the given step (WW → WWN) and the resultant DIM (c). The 20-dimensional vector constructed from the matrix is used as a descriptor to numerically characterize the protein sequence (d).

This novel method introduces encoding of amino acid sequences from the perspective of the decagonal isometries group (D_10_). The D_10_ has 20 elements—10 rotations *O*_*n*_ (by *nπ*/5 degrees) and 10 symmetries *S*_*n*_. A one-to-one correspondence can therefore be established between the elements of the group and the 20 amino acids. Our first step is arbitrarily assigning each element of D_10_ to an amino acid. Figure 2(a) presents the assignment of the amino acids to the elements of D_10_. Next, we identify an arbitrary edge of a decagon with the number 0 and subsequently put numbers 1 to 9 on consecutive edges.

Before we start coding our protein sequence, the initial position of the decagon is set such that the edge labeled 0 is at the bottom. The sequence is then inductively encoded by applying the transformations indicated by the group elements that correspond to consecutive amino acids in the sequence (Figure 2(c)). At each step, we look at the edge *n* that lies at the bottom of the decagon. For example, encoding WW → WWN requires the *S*_1_ symmetry transformation bringing the edge 5 of the decagon at the bottom (Figure 2(c)). The exact formulas of the said transformations are as follows:

(1) $$\begin{array}{c} O_{n}\left(X\right)=\left(X+n\right)\;\mathrm{mod}\;10,\;\text{for}\;n\text{th}\;\text{rotation},\;\\ S_{n}\left(X\right)=\left(10+n-X\right)\;\mathrm{mod}\;10,\;\text{for}\;n\text{th}\;\text{symmetry}.\;\; \end{array}$$

In theory, the number of times each edge lies at the bottom can be represented in a 10-dimensional vector to characterize the protein sequence. However, it would result in overcondensation of data. In order to reduce the loss of information, instead of considering just the bottom edge, at each step we also consider the edge to the right of the obtained bottom edge. A 10 × 10 decagonal isometries matrix (DIM) is thus constructed with the value *a*_*i*,*j*_ being the number of times that “*i*”-edge appears at the bottom of the decagon with “*j*”-edge at its right while applying transformations to the decagon considering a given sequence. Notice that DIM can have nonzero values only right above or right below the diagonal (with the exception of *a*_9,0_ and *a*_0,9_ entries). This allows us to transform DIM into 20-dimensional vector by putting all potentially non-zero values in a fixed order. The 20-dimensional vector finally acts as descriptors for the transmembrane protein segments encoded.

### 2.3. Representing Transmembrane Regions

The amino acid adjacency matrix and decagonal isometries matrix are used independently to encode the transmembrane and nontransmembrane protein segments. The associated matrix invariants mathematically characterize each of the membrane protein segments. Both representations are implemented independently and are used to distinguish between the transmembrane and non-transmembrane segments of membrane spanning proteins.

For this purpose, the transmembrane protein sequences are segmented into the transmembrane and non-transmembrane regions. The non-transmembrane regions are further divided into polypeptide segments of length 20 residues. It is essential to have the length of the non-transmembrane similar to that of the transmembrane segments in order to ensure better training of the classification models. All the transmembrane and non-transmembrane regions are then independently encoded using AA matrix and DIM. The encoded segments are divided into training and test sets.  Table 1 lists the number of particular segments in each set.

We perform principal component analysis (PCA) with the descriptors derived from AA matrix to check if the numerical descriptors are able to discriminate the transmembrane segments from the non-transmembrane ones. As PCA is projection of multidimensional data onto a coordinate system defined by the principal components, it gives an initial validation regarding choice of descriptors. Next, two independent counter propagation neural network (CPNN) models are developed using the invariants from both the matrices to distinguish between the transmembrane and non-transmembrane segments of the protein sequences.

## 3. Results and Discussion

### 3.1. Amino Acid Adjacency Matrix

To check if the row sum vector derived from the AA matrix well characterizes the transmembrane segments numerically, we perform the principal component analysis (PCA) and develop a CPNN model. 

**Figure 3:**
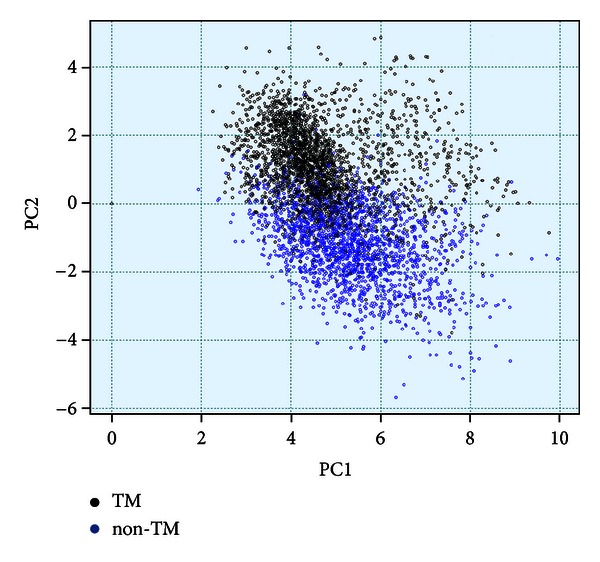
Principal component analysis. The transmembrane (black) and nontransmembrane (blue) segments form two different clusters.

Figure 3 shows the results from PCA analysis, where the transmembrane and non-transmembrane data are projected on 2D space defined by their first two principal components. PC1 contains 56.05% of the total variance, whereas PC2 contains 5.52% of the remaining variance. In total, the first two principal components contain 61.57% of the total variance present in the data. As we can see, the transmembrane and non-transmembrane segments, represented by the black and blue circles, respectively, are well separated over the first and second principal components. The region of overlap between the two clusters is very small with an overall distinction between the two groups. The PCA analysis is performed as a preliminary test. We have validated that the mathematical descriptors chosen are important to bring out the characteristic features of the protein segments. The descriptors are able to represent and distinguish the sequence characteristics of the two types of protein segments and group them successfully.

Next, we have developed a CPNN model to classify the protein segments as transmembrane or non-transmembrane ones. The model is optimized for both the training and the test sets simultaneously varying different network parameters. The goal is to obtain the optimal network parameters that minimize misclassification. In the final step, the optimized network is tested for its recall and prediction ability.

**Figure 4:**
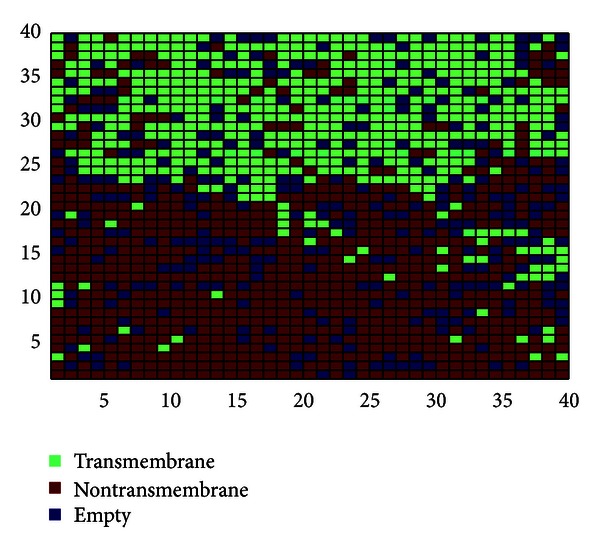
Top map of the optimized network. The transmembrane (green) and nontransmembrane (brown) segments form two different clusters. Empty neurons are dark blue.

The following network parameters are found to be optimal: network size—40 × 40, number of epochs—500, and maximum correction factor—0.9.  Figure 4 shows the top map of the optimized network with the transmembrane and non-transmembrane segments in two distinct clusters. The network shows only 4.33% error in recall ability; that is, it is able to correctly classify 95.67% of the segments in the training set. For the test set, the error is 8.67%.  Table 2 presents the detailed results of CPNN network.

### 3.2. Decagonal Isometries Matrix

The 20-dimensional vectors, derived from the decagonal isometries matrix, represent the transmembrane and non-transmembrane segments. The mathematically encoded protein segments in the training and test sets are then used to train and optimize a CPNN network. The goal is to optimize the network such that it is able to classify the two different types of protein segments based on the DIM invariant. 

The optimized network has the following configuration: network size—40 × 40, number of epochs—500, and maximum correction factor—0.5. The network shows 14.3% error in recall ability and 27.1% error in prediction ability, with error threshold at 0.501. The detailed results are given in Table 3.

### 3.3. Advantage of Mathematical Characterization

Analyzing the protein sequence is the first step towards determining its structure and function. With the growing number of proteins sequenced, there is a necessity of novel techniques to characterize the sequences. Presently, most commonly used protein sequence descriptors are based on evolutionary information and physiochemical properties. Even though these methods have proved to be efficient in most cases, in certain special cases like that of transmembrane proteins, they may fall short. As the vast field of transmembrane proteins largely remains unexplored with many transmembrane proteins yet to be sequenced, it is possible to obtain new protein sequences without any known homologs. In such case, traditional sequence analysis methods based on alignment profiles would not be sufficient to analyze the novel sequences. The evolutionary information-based descriptors are therefore inadequate. As several indices of the same physiochemical property exist, such descriptors can cause ambiguity. Therefore, there is a need of developing novel methods based on sequence information alone to represent protein sequences.

The two matrix representations, amino acid adjacency matrix and decagonal isometries matrix, of the protein segments are derived from the sequence information. The physiochemical properties of the amino acids and the evolutionary information of the sequence based on alignment profiles are not utilized for characterizing. Moreover, the matrices are labeled with the amino acids arbitrarily. The mathematical descriptors are dependent on the sequence information alone and successfully reveal underlying characteristics and patterns of a given sequence. Such descriptors are useful in representing novel sequences independently. Their numerical nature also makes them easier to be incorporated into a mathematical model. In addition, one can derive different invariants to be used as descriptors from the same matrix representations depending on the problem to be addressed.

## 4. Conclusion

In this work, we have successfully used mathematical descriptors to characterize transmembrane regions of proteins. Amino acid adjacency matrix and decagonal isometries matrix are independently used to encode the protein segments. Both the representations are successful in revealing the sequence characteristics particular to a specific group of protein segments and in classifying them accordingly as transmembrane and non-transmembrane. The accuracy of the former method was better, which challenges the potential optimization and further development of the latter one. Depending only on the sequence information, the mathematical representations described here can prove to be powerful tool in developing novel sequence analysis methods, especially for less explored protein classes like transmembrane proteins.

## Figures and Tables

**Table 1:** Training and test sets.

Sets	Number of segments		
	Total segments	Transmembrane	Nontransmembrane
Training	4204	1867	2337
Test	450	200	250

**Table 2:** Classification model using amino acid adjacency matrix.

Sets	Network results		
	Total segments	Segments correctly classified	% error
Training	4204	4022	4.33
Test	450	411	8.67

**Table 3:** Classification model using decagonal isometries matrix.

Sets	Network results	
	Correlation coefficient (*r*∗*r*)	% error
Training	0.61	14.3
Test	0.255	27.1
